# Altered Epithelial-mesenchymal Plasticity as a Result of *Ovol2* Deletion Minimally Impacts the Self-renewal of Adult Mammary Basal Epithelial Cells

**DOI:** 10.1007/s10911-021-09508-0

**Published:** 2022-01-04

**Authors:** Peng Sun, Yingying Han, Maksim Plikus, Xing Dai

**Affiliations:** 1 Department of Biological Chemistry, School of Medicine, University of California, CA Irvine, 92697 USA; 2 Department of Developmental and Cell Biology, University of California, Irvine, CA 92697 USA

**Keywords:** Mammary gland, Basal cell, Stem cell, *Ovol2*, Epithelial-to-mesenchymal transition, Epithelial-mesenchymal plasticity

## Abstract

Stem-cell containing mammary basal epithelial cells exist in a quasi-mesenchymal transcriptional state characterized by simultaneous expression of typical epithelial genes and typical mesenchymal genes. Whether robust maintenance of such a transcriptional state is required for adult basal stem cells to fuel self-renewal and regeneration remains unclear. In this work, we utilized *SMA-CreER* to direct efficient basal cell-specific deletion of *Ovol2*, which encodes a transcription factor that inhibits epithelial-to-mesenchymal transition (EMT), in adult mammary gland. We identified a basal cell-intrinsic role of *Ovol2* in promoting epithelial, and suppressing mesenchymal, molecular traits. Interestingly, *Ovol2*-deficient basal cells display minimal perturbations in their ability to support tissue homeostasis, colony formation, and transplant outgrowth. These findings underscore the ability of adult mammary basal cells to tolerate molecular perturbations associated with altered epithelia-mesenchymal plasticity without drastically compromising their self-renewal potential.

**Supplementary Information:**

The online version contains supplementary material available at 10.1007/s10911-021-09508-0.

## Introduction

The mouse mammary gland is a dynamic and regenerative organ that undergoes most of its development after birth, with dramatic structural and/or functional changes occurring during puberty, estrus cycle, pregnancy, lactation, and involution [1, 2]. Under the regulation of hormones and local growth factors, stem/progenitor cells in the mammary epithelium self-renew, proliferate, and differentiate to drive the growth, remodeling, and regeneration of the bi-lineage epithelial network composed of an outer basal layer and an inner luminal layer [3]. The basal cells differentiate into myoepithelial cells that exhibit both epithelial and smooth muscle characteristics, and they contain unipotent and multi/bi-potent stem cell subsets that fuel morphogenesis and regeneration [3–10].

Epithelial-to-mesenchymal transition (EMT) is a form of cellular plasticity where epithelial cells lose or attenuate epithelial traits and gain mesenchymal traits [11–16]. It has become increasingly clear that EMT in vivo is not a binary process, but rather encompasses a heterogeneous spectrum of diverse intermediate or hybrid cellular states that exist between epithelial and mesenchymal phenotypes. Interestingly, mammary basal/myoepithelial cells express both epithelial and mesenchymal genes, and are in a “quasi-mesenchymal” transcriptional state that resembles intermediate cell states within the epithelial-mesenchymal spectrum [17–20]. In breast cancer cells, expression of core EMT-inducing transcription factors is linked to the acquisition of stem cell traits [21–26]. How the “quasi-mesenchymal” transcriptional state is maintained in adult mammary tissue basal cells and whether aberrant epithelial-mesenchymal plasticity (EMP) alters stemness remain to be fully understood.

We previously reported that deletion of EMT-suppressive transcription factor *Ovol2* in the entire mammary epithelium halts ductal morphogenesis during pubertal development [27, 28]. Here we use *SMA-CreER* to drive the deletion of *Ovol2* specifically in adult mammary basal cells, and find that this leads to significantly enhanced EMT-like molecular features. However, the *Ovol2*-deficient basal cells are not outcompeted by *Ovol2*-intact basal cells during tissue homeostasis, and are capable of regenerating new mammary trees upon transplantation and producing colonies in vitro. Interestingly, acute deletion of *Ovol2* in cultured basal cells results in increased colony formation. Together, these findings reveal a context-dependent role of *Ovol2* in basal cell proliferation/self-renewal and the robustness of adult mammary basal cells in tolerating EMT-like gene expression perturbations.

## Results

### *SMA-CreER* Directs Inducible, Efficient, and Specific Gene Deletion in Adult Mammary Basal Cells

**Fig. 1:**
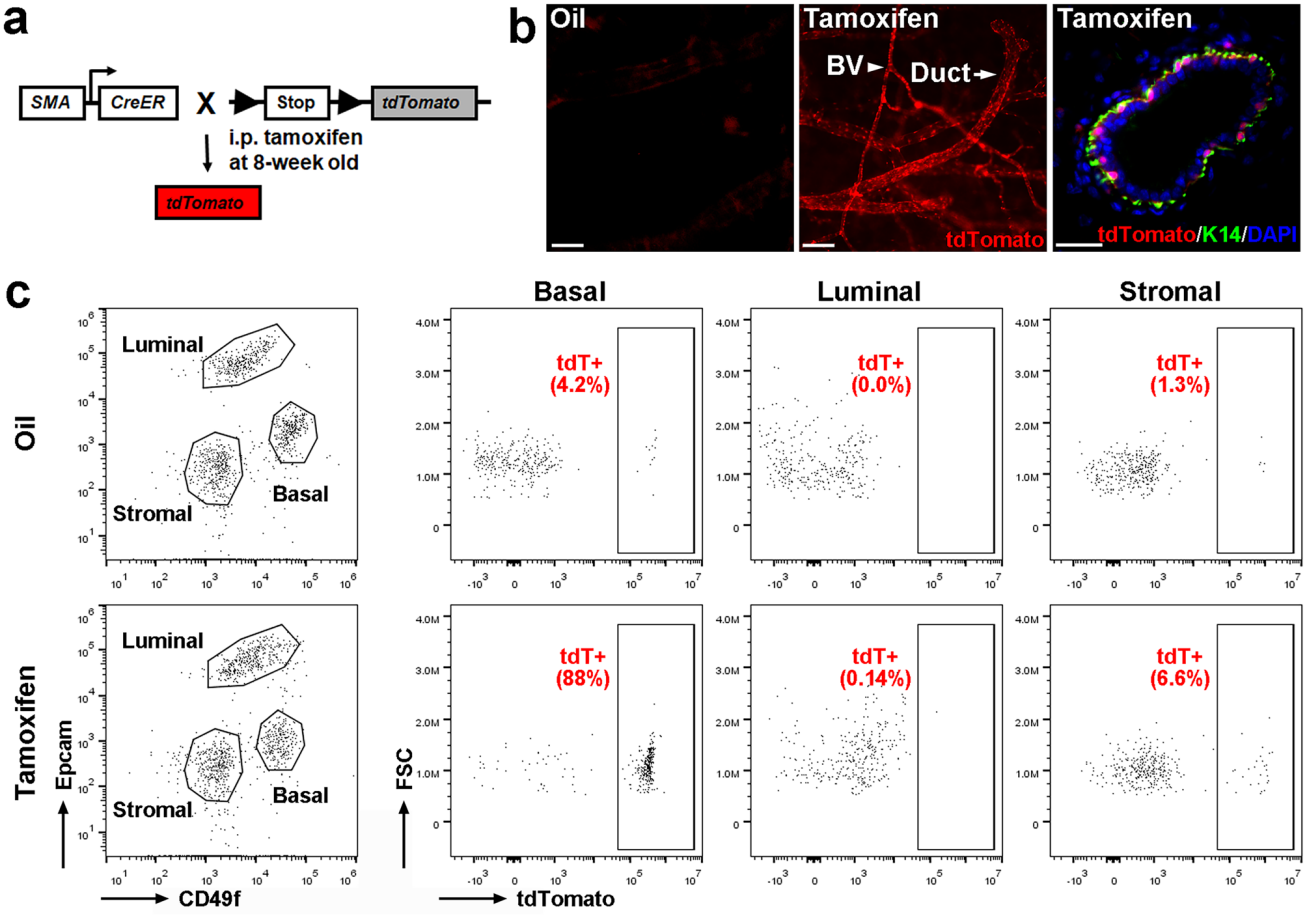
*SMA-CreER* directs basal cell-specific recombination in adult mammary gland. **a**, Experimental design. **b**, Microscopic analysis of tdTomato fluorescence in whole-mount preparation (middle) and section (right) of mammary glands from *SMA-CreER;tdTomato* females at 14 days after tamoxifen injection. Oil was used as a negative control (left). BV, blood vessel. Scale bar: left and middle, 100 μm; right, 25 μm. **c**, Representative flow cytometry profiles of immunostained cell lineage surface markers (left) and tdTomato (tdT) fluorescence (right). FSC, forward scatter. Mammary glands were harvested at 14 days after injection

*SMA-CreER* has been used to lineage-trace mammary basal cells [7], but its utility in directing basal cell-specific gene deletion for functional studies has not been explored. Towards that end, we bred *SMA-CreER* mice with *tdTomato* mice, and treated the resulting *SMA-CreER;tdTomato* females with two doses of tamoxifen in adulthood (Fig. 1a). Two weeks after treatment, we detected robust tdTomato fluorescence in mammary ducts and blood vessels using whole-mount microscopy (Fig. 1b, middle). Immunostaining with K14 antibody to mark basal cells revealed nuclear tdTomato expression in the basal layer (Fig. 1b, right). Flow cytometry showed that > 80% of the basal cells express tdTomato, whereas less than 1% of the luminal cells and ~ 7% of the stromal cells are tdTomato^+^ (Fig. 1c). These data show that *SMA-CreER* is a useful tool for adult mammary basal cell-targeted gene deletion.

### *Ovol2* Acts Cell-autonomously Within the Basal Layer to Promote Epithelial, and Suppress EMT-associated, Gene Expression

**Fig. 2:**
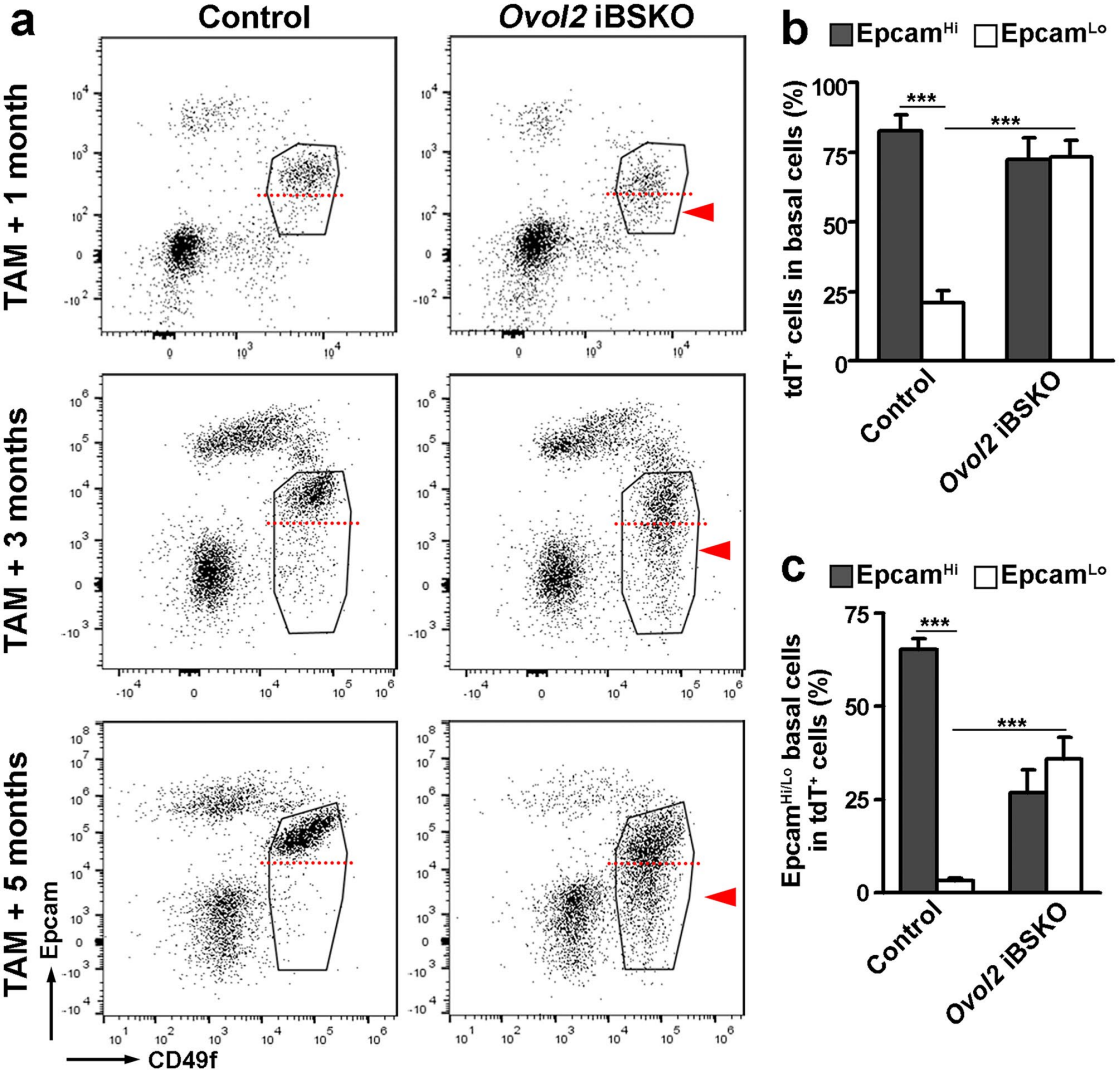
Emergence of Epcam^low^ basal cells in *Ovol2* iBKSO mice. **a**, Representative flow cytometry profiles of mammary cells at the indicated time points after tamoxifen induction. Red arrowheads point to Epcam^low^ basal cells, demarcated by dashed red lines from Epcam^high^ basal cells. **b**, Quantitative analysis of tdTomato-positive cells in Epcam^low^ and Epcam^high^ basal cells at three months after tamoxifen injection. **c**. Quantitative analysis of Epcam^low^ and Epcam^high^ cells in tdTomato-positive cells at three months after tamoxifen injection. n = 5 pairs of mice in (**b**) and (**c**). *** *p* < 0.005. tdT, tdTomato. Control (Ctrl) genotype: *SMA-CreER;Ovol2*^*f/*+^*;tdTomato*

Towards assessing the function of *Ovol2* specifically in the mammary basal layer, we used *SMA-CreER* mice to inducibly delete *Ovol2* in adulthood. *SMA-CreER;Ovol2*^*f/−*^ (inducible, basal cell-specific knockout or iBSKO) mice were generated and treated with tamoxifen at eight weeks of age along with control littermates. With time, the basal cells in iBSKO mice exhibited visible reduction in surface level of epithelial marker protein Epcam (Fig. 2; Online Resource 1a). Importantly, Epcam^low^ basal cells were detected only with tamoxifen injection, but not with oil injection (Online Resource 1b), indicating that their emergence is dependent on *Ovol2* deletion. The relative frequencies of basal and luminal cells were not affected (Online Resource 1c).

**Fig. 3:**
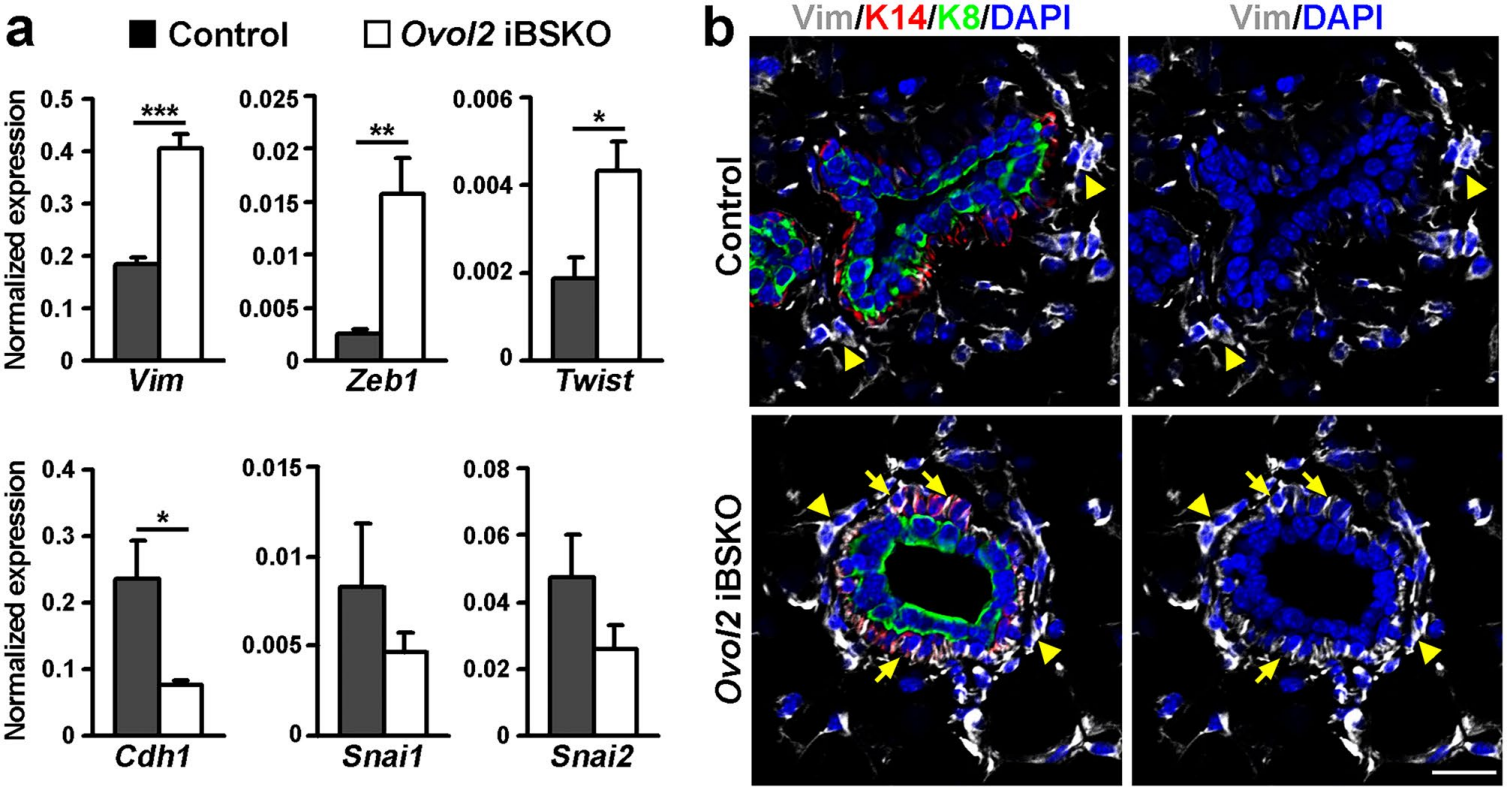
Molecular changes in *Ovol2* iBKSO basal cells suggests a partial epithelial-to-mesenchymal drift. **a**, RT-qPCR analysis of the indicated genes. n = 4 for *Zeb1* and n = 3 for *Vim*, *Twist*, *Cdh1*, *Snai1* and *Snai2*. *** *p* < 0.005, ** *p* < 0.01, * *p* < 0.05. **b**, Immunofluorescence of vimentin protein. K14 and K8 antibodies stain basal cells and luminal cells, respectively. DAPI stains the nuclei. Arrows and arrowheads point to vimentin-positive basal cells and stromal cells, respectively. Scale bar: 25 μm. Mammary glands were harvested at three months after tamoxifen injection. Control genotype: *SMA-CreER;Ovol2*^*f/*+^*;tdTomato*

To determine if basal-specific *Ovol2* deletion affects EMT-associated gene expression, we FACS-sorted basal cells at three months after tamoxifen induction, and performed RT-qPCR analysis. *Ovol2* iBKSO basal cells showed significantly increased expression of mesenchymal gene *Vim* (encoding vimentin), as well as of *Zeb1* and *Twist*, which encode EMT-inducing transcription factors (Fig. 3a). In contrast, the expression of epithelial adhesion gene *Cdh1* was significantly decreased in *Ovol2* iBKSO basal cells, whereas the expression of other EMT-inducing transcription factors *Snai1* and *Snai2* was not affected (Fig. 3a). Immunofluorescence also detected more prominent vimentin protein expression in the mammary basal, but not luminal, cells of iBSKO mice compared to control littermates (Fig. 3b). Together, these data show that *Ovol2* is required in the adult basal cells to promote the epithelial-associated, and suppress the EMT/mesenchymal-associated, molecular traits, thereby maintaining a quasi-mesenchymal transcriptional state and keeping EMT-like changes in check.

### Basal Cell-specific Deletion of *Ovol2* Does Not Impact Mammary Homeostasis

**Fig. 4:**
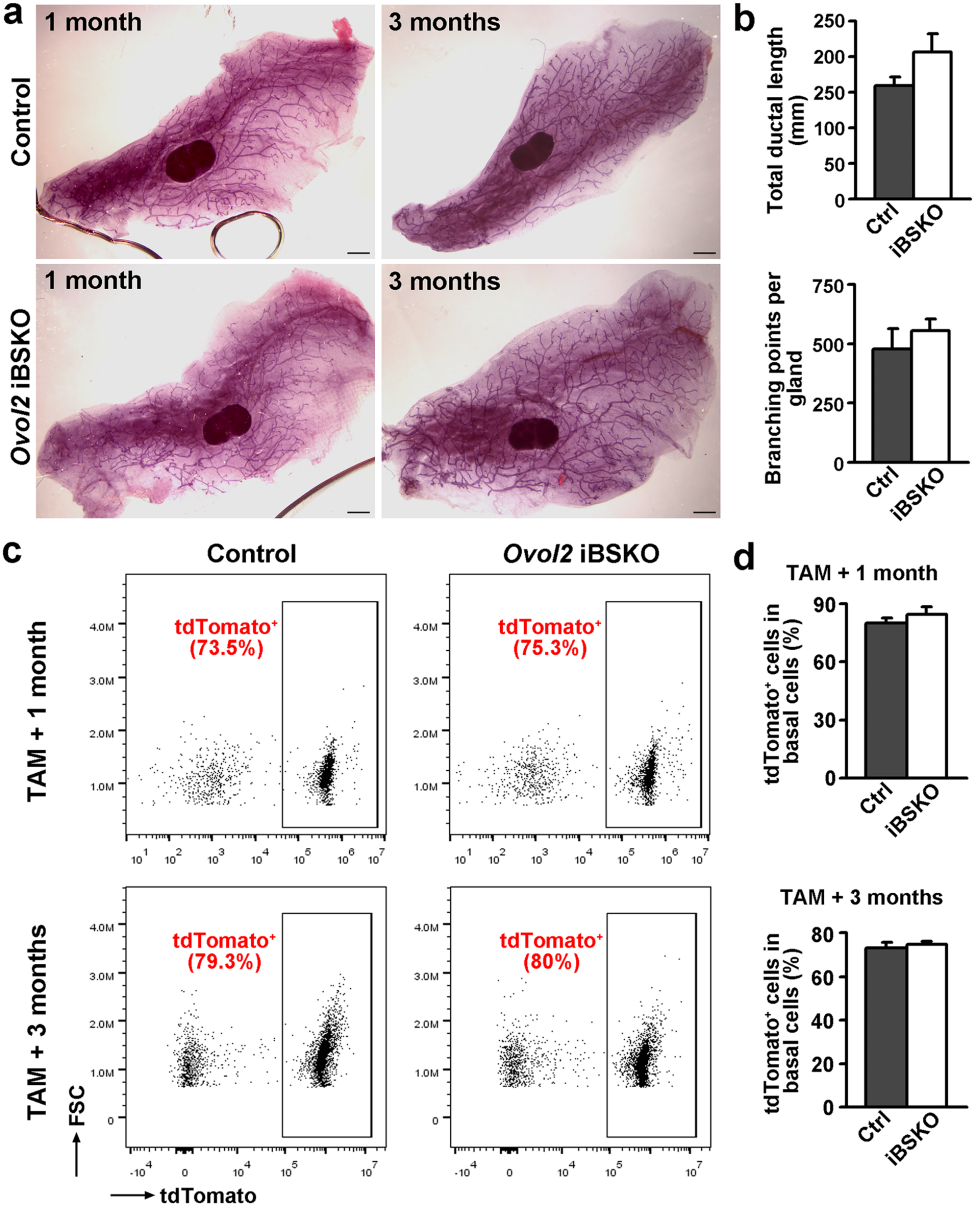
*Ovol2* loss from adult basal cells does not alter mammary gland homeostasis. **a**, Representative whole-mount carmine staining images of mammary glands from control and *Ovol2* iBSKO littermates at the indicated times after tamoxifen injection. Scale bar: 1 mm. **b**, Quantitative analysis of ductal length and branching at three months after tamoxifen injection. n = 3 pairs of mice. **c**, Representative flow cytometry profiles to show frequency of tdTomato-positive cells in control and *Ovol2* iBSKO basal cells at the indicated times after tamoxifen injection. **d**, Quantitative analysis of tdTomato-positive basal cells as in (**c**). *n* = 3 pairs of mice for one month and *n* = 6 pairs for three months of age. FSC, forward scatter. Control (Ctrl) genotype: *SMA-CreER;Ovol2*^*f/*+^*;tdTomato*

To examine the functional impact of *Ovol2* loss-induced alteration in basal EMP, mammary gland morphology of *Ovol2* iBSKO and control littermates was analyzed at one month and three months after induction of gene deletion. No significant difference was observed in either ductal length or branching complexity (Fig. 4a, b). iBSKO and control littermates were also bred at one month after tamoxifen induction, and their pups were found to gain weight similarly (Online Resource 2), implying that lactation may not be dramatically affected by *Ovol2* deletion in the basal cells.

To ask if the residual *Ovol2*-intact basal cells in *Ovol2* iBSKO mice preferentially contribute to mammary epithelial homeostasis, we performed flow cytometry to compare the percent of tdTomato^+^ (i.e., *Ovol2*-deleted) basal cells in control and iBSKO mice that also contain the *tdTomato* allele at one month and three months after tamoxifen treatment. At both times, tdTomato^+^ basal cells were present at comparable frequencies in mammary glands from control and iBSKO mice (Fig. 4c, d). Moreover, the percent of tdTomato^−^ (i.e., *Ovol2*-intact) did not increase over time in iBSKO mice (Fig. 4c, d). These data argue against the possibility that residual *Ovol2*-intact basal cells outcompete *Ovol2*-deficient basal cells during adult tissue homeostasis.

### *Ovol2* Expression in Basal Cells is Largely Dispensable for Self-renewal and Regeneration

**Fig. 5:**
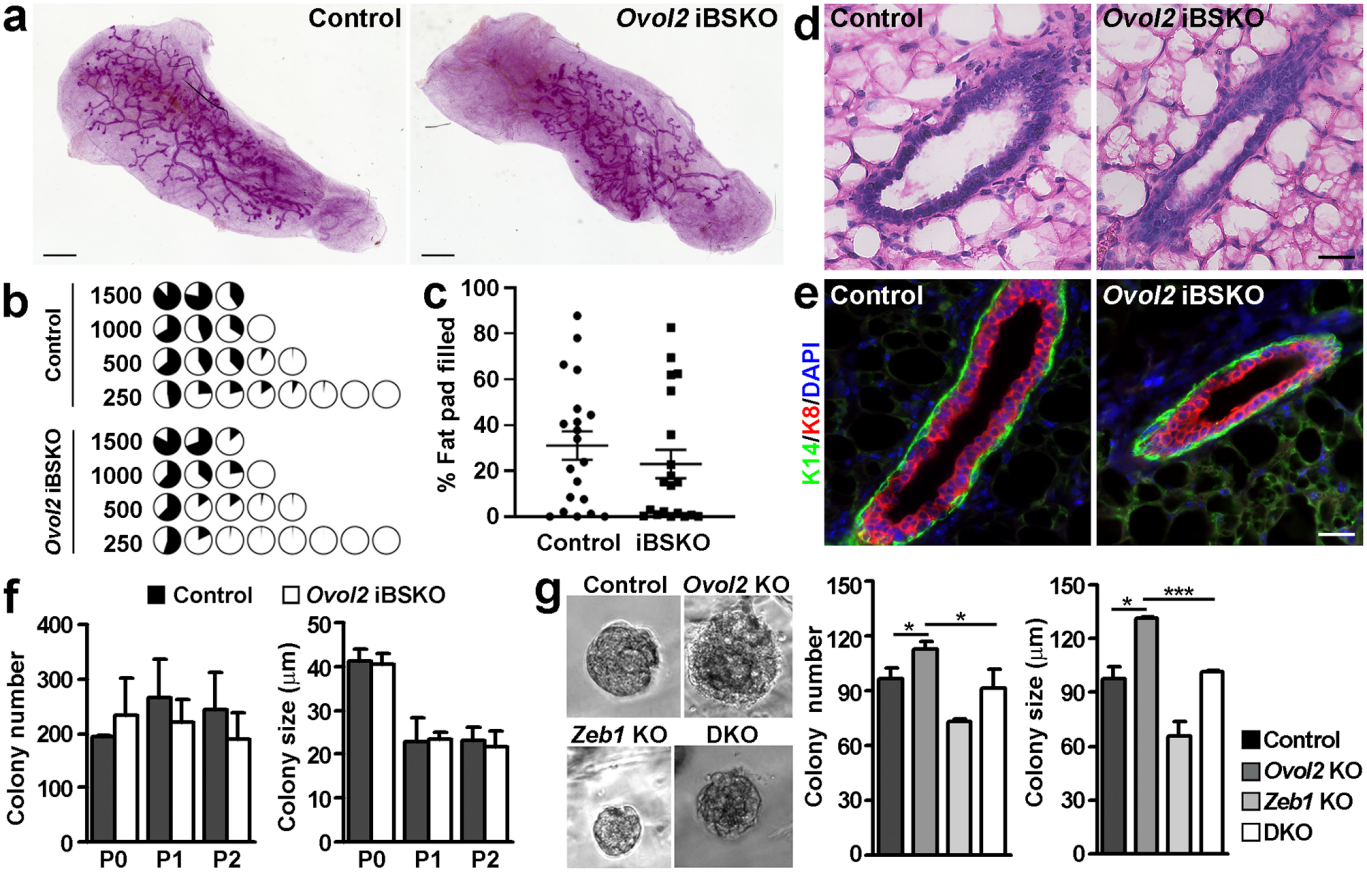
*Ovol2*-deficient basal cells show largely normal regenerative potential and colony formation. **a**, Representative transplants derived from tdTomato^+^ basal cells isolated from control and *Ovol2* iBSKO littermates at one month after tamoxifen injection. Scale bar: 1 mm. **b**-**c**, Summary of limiting dilution transplantation results using 3 pairs of donor mice. Each pie diagram (**b**) represents an outgrowth, and data for percent of fat pad filled are summarized in (**c**). A tree that is > 5% of the fat pad indicates a positive take. Each experiment included 1 pair of *Ovol2* iBSKO and control littermate as donor mice, and basal cells from each were transplanted at the indicated number onto the contralateral sides of the same host mice. **d**-**e**, H/E staining (**d**) and indirect immunofluoresence (**e**) of the transplants. Scale bar: 25 μm. **f**, Colony formation by control and *Ovol2* iBKSO basal cells at one month post-tamoxifen injection. P0, initial plating. Colonies from P0 were harvested and equal numbers of control and iBKSO cells were subsequently passaged once (P1) or twice (P2). Shown are summary of 3 independent experiments. **g**, Colony formation by control (Ade-Cre-infected *Ovol2*^+/f^*;Zeb1*^+/f^), *Ovol2* single (Ade-Cre-infected *Ovol2*^f/f^*;Zeb1*^+/f^), *Zeb1* single (Ade-Cre-infected *Ovol2*^+/f^*;Zeb1*^f/f^), and *Ovol2/Zeb1* double (D; Ade-Cre-infected *Ovol2*^f/f^*;Zeb1*^f/f^) knockout (KO) basal cells. n = 3 for each. Represent colony images are shown on the left, and summary of three independent experiments using different mice are shown on the right. *** *p* < 0.005, * *p* < 0.05

To examine whether *Ovol2* loss from the basal cells impacts their stem cell function, we conducted transplantation experiments by injecting FACS-sorted tdTomato^+^ basal cells from *Ovol2* iBSKO and control littermates into de-epithelialized fat pads of congenic 3-week-old host mice. Using serially decreasing numbers of basal cells, fat pad filling by *Ovol2*-deficient basal cells was overall similar to that by control basal cells (Fig. 5a–c; Online Resource 3a). The overall take rate for all transplants analyzed was 85% (17/20) for control and 80% (16/20) for *Ovol2*-deficient basal cells (Fig. 5b). We also analyzed the epithelial structure of the transplants using H/E staining and immunofluorescence, which revealed largely normal ductal histology and expression of basal (K14) and luminal (K8) keratin markers (Fig. 5d, e). These results suggest that *Ovol2* expression in adult basal cells is not essential for the self-renewal and regenerative potential of these cells.

To complement the in vivo findings above, we next performed ex vivo 3D-Matrigel colony formation assays by culturing sorted tdTomato^+^ basal cells from *Ovol2* iBSKO (enriched for *Ovol2*-deficient cells) and control littermates. Colonies formed by the *Ovol2* iBSKO basal cells were similar in number and size to those formed by control cells both in initial plating and when the cells were serially passaged (Fig. 5f; Online Resource 3b). To ask whether *Ovol2* may play a role in adult basal cell proliferation/self-renewal that only manifests under certain conditions, we sorted basal cells from adult mice containing floxed *Ovol2* alleles (*Ovol2*^f/f^), infected them with Cre-expressing adenoviruses (Ade-Cre) to acutely delete *Ovol2* [29], and analyzed colony formation. We found that this led to a slight but significant increase in colony number and size compared to the *Ovol2*-intact control (Fig. 5g). Interestingly, simultaneous deletion of *Zeb1*, an EMT-promoting factor that we previously shown to be an Ovol2 target in developing mammary epithelium [28], normalized this increase (Fig. 5g). Taken these data together with our in vivo findings, we surmise that *Ovol2*’s role in adult mammary basal cell proliferation/self-renewal is context-specific but centers around the inhibition of EMT-associated gene expression.

## Discussion

The acquisition of stem-like traits by breast cancer cells through ectopic expression of EMT-inducing factors has led to the suggestion that EMT-like plasticity may also confer stemness to normal epithelial cells [20, 22–24]. However, lineage tracing experiments in vivo has revealed that mammary basal cells expressing elevated levels of EMT-associated genes (e.g., *S100a4, Zeb1*) actually show reduced regenerative potential than other basal cells [19]. Our work adds clarification to this issue of debate by showing that adult mammary basal cells with attenuated molecular epithelial traits and enhanced molecular mesenchymal traits, as a consequence of losing epithelial-promoting transcription factor Ovol2, exhibit similar in vivo transplantability as their Ovol2-expressing counterparts. Thus, certain alterations in basal cell EMP can be uncoupled from functional manifestation of stem cell self-renewal.

Interestingly, colony formation of *Ovol2*-deficient cells in vitro is variably affected depending on the exact method of deletion, with *SMA-CreER*-directed deletion in vivo leading to no change whereas Ade-Cre-directed deletion in vitro leading to a detectable increase. As such, *Ovol2* shows a context-dependent requirement in adult basal cell function.

Our findings also provide new insights into the difference and similarity between developmental and adult mammary basal cells in their EMP tolerance. *K14-Cre*-driven pan-epithelial-deletion of *Ovol2* almost completely arrests pubertal ductal morphogenesis, and FACS-sorted *Ovol2*-deficient basal cells from these mice cannot efficiently regenerate mammary trees upon transplantation [28]. In contrast, basal-specific *Ovol2* deletion does not significantly compromise or enhance regenerative potential at least under the conditions of our experiments (this work). In both cases, however, *Ovol2* loss results in detectable changes in the expression of EMT-associated genes, indicating a shared requirement by developmental and adult basal cells for *Ovol2* to robustly maintain their transcriptional programs in a quasi-mesenchymal, but still epithelial, state.

How to explain the differential responses to altered EMP by the developmental vs. adult basal cells as well as by the adult basal cells in which *Ovol2* is deleted using *SMA-CreER* or Ade-Cre? The extent and duration of EMT-like changes might be critical. The relatively slow turnover of adult basal cells during homeostasis, compared to their developmental counterparts during pubertal development, may implicate a smaller demand for EMP maintenance. Functional perturbations to adult basal cells may manifest only after the cells are in an EMT-like state for more extensive periods of time. Unfortunately, we were unable to efficiently transplant basal cells from both WT and *Ovol2*-deficient mice that are five months of age (data not shown), preventing us from experimentally testing this possibility. The precise cell adhesion status of *Ovol2*-deficient basal cells may also influence basal cell proliferation/self-renewal. As such, extensive experimental manipulation in culture may have altered cell adhesion to the degree that unmasks an advantageous effect of enhanced EMT-like features in adult basal cells. Lastly, we cannot fully exclude the possibility that the tdTomato-positive basal cell population still contains residual *Ovol2*-intact cells because of differential Cre-mediated recombination efficiency at different loci, and these cells may have also contributed to regeneration in vivo and colony formation in vitro.

Another important difference between the pan-epithelial *Ovol2* knockout and basal cell-specific *Ovol2* knockout models is that *Ovol2* is also deleted in luminal cells of the former. Loss of *Ovol2* may compromise the function of luminal cells, which in turn influence basal cell function, explaining the severe defects in mammary epithelial development and regeneration in the pan-epithelial mutant mice. Of interest, organoids produced by terminal end buds isolated from pan-epithelial *Ovol2* knockout mice contain almost entirely K14-positive (basal) cells but few K18-positive (luminal) cells (data not shown), suggesting a possible role for *Ovol2* in promoting luminal differentiation. Whether this function occurs at the binary junction between basal and luminal cells or entirely in luminal cells will be an interesting future pursuit. Our observation that basal cell-specific deletion of *Ovol2* does not affect basal/luminal cell frequencies seems to suggest the latter. This said, it is also possible that *SMA-CreER*, while proven by our work to be effective in directing gene deletion in bulk basal cells during adulthood, may not efficiently target the specific basal cell subsets that express low levels of *Acta2* but are already destined to a luminal fate.

In summary, our work uncovers a functional regulator (*Ovol2*) of adult basal cell EMP, but at the same time underscores the ability of basal cells to tolerate molecular perturbations associated with EMT enhancement without drastically compromising their self-renewal potential. These findings may also inform our understanding of the mechanistic connection between EMT and stemness of basal-like cells in breast cancer.

## Methods

### Mice

*SMA-CreER* and *Ovol2*^f/f^ mice were as previously reported [7, 28]. *ROSA*^*tdTomato*^ mice were purchased from the Jackson Laboratory (Stock # 007909). Primers used for genotyping are listed in Table S1. All mouse experiments have been approved by and conform to the regulatory guidelines of the Institutional Animal Care and Use Committee of the University of California, Irvine.

### Tamoxifen Treatment

Tamoxifen (Sigma, T5648) was prepared at a concentration of 20 mg/ml in corn oil (Sigma, C8267). Eight-week-old mice received two intraperitoneal (IP) injections (24 h apart) of 2 mg of tamoxifen per 20 g body weight or corn oil (100 µl per 20 g body weight). Mammary glands were harvested at 24 h to five months after the second injection.

### Isolation of Mammary Epithelial Cells

Mammary cell preparations were performed as previously described [28, 30, 31]. Briefly, mammary glands were isolated from female mice and placed in the digestion mix [DMEM/F12 (1:1) with 5% fetal bovine serum (FBS) (Omega Scientific, FB-02), 300 U/mL collagenase (Sigma, C9891) and 100 U/mL hyaluronidase (Sigma, H3506)] for 1.5 h at 37 °C. Cells were pelleted and resuspended in red blood cell lysis buffer (Sigma, R7757). Single cell suspension was obtained by further treatment with 0.25% trypsin–EDTA (Gibco, 25200), 10 mg/mL DNase (Sigma, DN25), and 5 mg/mL dispase (Stem Cell Technologies, 07913), followed by filtration using a 40 μm-pore mesh filter (SWiSH, TC70-MT-1).

### Cell Labeling and Flow Cytometry

Single cell suspension from above was stained using the following antibodies and reagents: anti-CD49f-FITC (1:250, Bio Legend, 102205), anti-EpCAM-PE-Cy7 (1:250, Bio Legend, 118215), anti-lineage-APC [1:250; including APC-CD45 (BD Biosciences, 559864), APC-CD31 (BD Biosciences, 551262), APC-TER119 (BD Biosciences, 557909)], and SytoxBlue (Invitrogen, S3457). Flow cytometry analysis and sorting were performed on a Novocyte and FACSAria (Becton Dickenson UK), respectively.

### RT-qPCR Analysis

Total RNA was extracted from FACS-sorted cells using RNeasy Mini Kit with on-column DNase treatment according to manufacturer’s protocol (Zymo Research, R1050). cDNA was synthesized using High-Capacity cDNA Reverse Transcription Kit (Thermo Fisher Scientific, 4368814) according to manufacturer’s instructions. Real-time PCR was performed using a SYBR Green Supermix (BioRad, 1725122) on a CFX96 RT-qPCR system, and data were analyzed using the 2 − ΔΔCT method. Primers used for qPCR are listed in Table S1.

### Immunohistochemistry and Mammary Gland Whole-mount Analysis

Immunohistochemistry was performed as previously described [28, 30, 31] using the following antibodies: vimentin (rabbit, Cell Signaling, 5741P), K14 (chicken or rabbit, a gift from J. Segre, National Institutes of Health, Bethesda, MD) and K8 (rat, Developmental Studies Hybridoma Bank).

For whole-mount analysis, the #4 pair of mammary glands were dissected and fixed in Carnoy’s fixative (10% acetic acid, 30% CHCl_3_, 60% ethanol) for 2–4 h. Fixed tissues were treated with a gradient of ethanol (100%, 70%, 30%) and then washed with sterile water for 10 min. Tissues were then incubated with carmine-alum staining solution as previously described. Images were captured using a Keyence microscope.

### Cleared Fat Pad Transplantation

Fat pad clearing and transplantation was as previously described [28, 30, 31]. Briefly, single cell suspensions of sorted tdTomato-positive basal cells from *Ovol2* iBSKO and control littermates were prepared as described above and diluted in a 1:1 solution of 5% FBS media/Matrigel at limiting dilutions (2,000, 1,500, 1,000, 500, and 250 cells per 10 µl). Ten μL of the cell/Matrigel solution was injected into cleared fat pads of #4 mammary glands of 3-week-old C57BL/6 females, with each host mouse receiving contralateral injections of *Ovol2* iBSKO and control samples. Outgrowths were analyzed 8–9 weeks later using whole-mount carmine alum staining.

For histology and immunostaining of carmine-stained mammary outgrowths, mammary whole-mounts were immersed in xylene to remove excess permount and processed in paraffin overnight before embedding. The embedded outgrowths were then sectioned, and stained with H/E or K8/K14 antibodies using established methods [32, 33].

### 3D-matrigel Colony Assay and Ade-Cre Infection

FACS-sorted tdTomato-positive basal cells from *Ovol2* iBSKO and control littermates were resuspended in a chilled 1:1 solution of EpiCult-B medium (Stem Cell Technologies, 05610)/growth factor-reduced Matrigel (BD Biosciences, CB-40230), and 4000 cells were plated into one well from 8-well chamber slides (Thermo Fisher Scientific, 154534). After Matrigel hardens, 400 μl of EpiCult-B medium containing 10 ng/mL EGF (Millipore, 01–107), 10 ng/mL bFGF (PeproTech, 100-18B), and 4 μg/mL heparin (Stem Cell Technologies, 07980) was added into each well. Culture medium was changed every three days for two weeks, followed by counting colony number and measuring colony size. To passage the colonies, medium was first removed and Matrigel dissolved using 200 μL of dispase for at least 20 min. Single cells were obtained by incubation in 0.25% Trypsin–EDTA followed by filtration using a 40 μm filter. Cells were then re-plated as described above.

GFP-expressing Ade-Cre viruses were purchased from Vector Biolabs. FACS-sorted basal cells were plated at 10 million/mL/well in 24-well plates in DMEM/F12 (Stemcell Technologies, 36254) containing 2% FBS, 10 mM HEPES (Millipore Sigma, H3375), 10 ng/ml EGF (Invitrogen, PMG-8043), 250 ng/ml Rspo1 (R&D, 3474-RS), 100 ng/ml Noggin (Fisher Scientific, 50–399-006), and 10 μM Rock inhibitor Y27632 (Millipore Sigma, SCM075), and cultured overnight. Cells were then infected with Ade-Cre at a multiplicity of infection (MOI) of 10 for overnight in 24-well ultra-low attachment surface polystyrene plate (Corning, REF 3473). Cells were harvested the next day, and treated with 400 μL TrypLE Select (GIBCO, 12605–010) for 20 min at 37 °C followed by neutralization with 2 mL HBSS buffer (GIBCO, 14025134). GFP^+^ cells were FACS-sorted for colony formation assay.

### Statistics

Experiments were performed on at least 3 biological replicates or repeated at least twice. The sample size and number of independent experiments are indicated in the relevant figure legends. For analysis of differences between groups, Student’s unpaired *t*-test was performed with 2-tailed in Excel. *p* values of 0.05 or less were considered statistically significant. Error bars represent mean ± SEM.

## Supplementary Information

Below is the link to the electronic supplementary material.

Supplementary file1 (TIF 12714 KB)

Supplementary file2 (TIF 2617 KB)

Supplementary file3 (TIF 8468 KB)

Supplementary file4 (XLSX 11 KB)
