# Supplementary figures and images for: Altered Epithelial-mesenchymal Plasticity as a Result of Ovol2 Deletion Minimally Impacts the Self-renewal of Adult Mammary Basal Epithelial Cells
Source: J Mammary Gland Biol Neoplasia. 2022 Jan 4;26(4):377–86. doi: 10.1007/s10911-021-09508-0 (PMC8858298; doi:10.1007/s10911-021-09508-0)

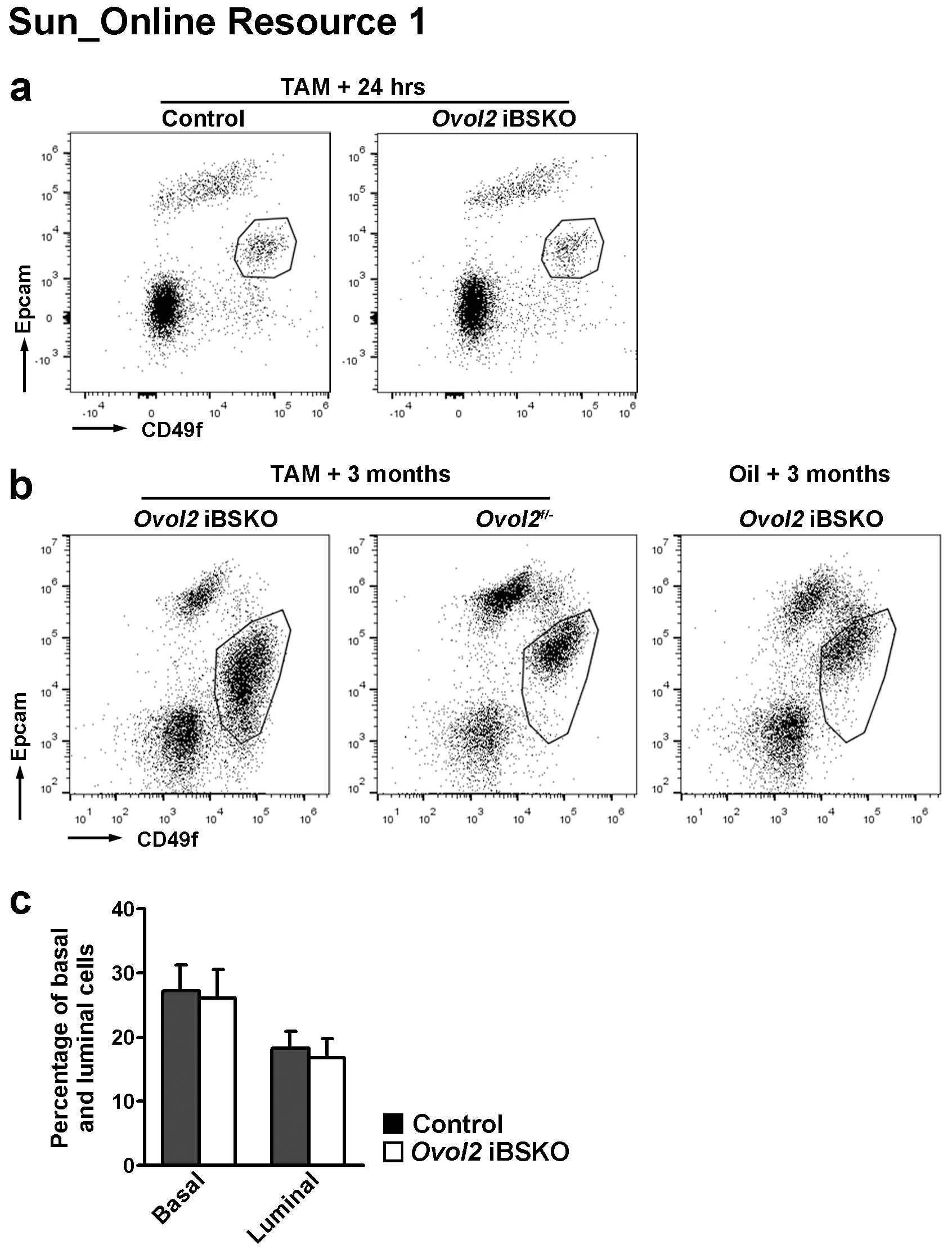

Supplement: Supplementary file 1 — Supplementary file1 (TIF 12714 KB) [file 10911_2021_9508_MOESM1_ESM.tif]

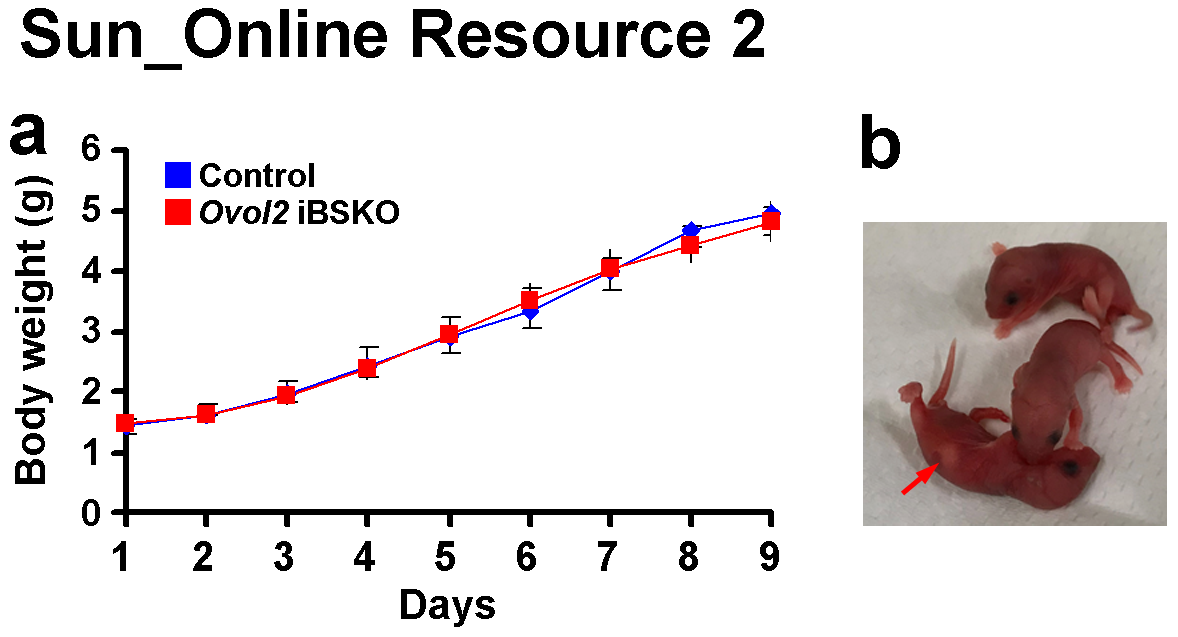

Supplement: Supplementary file 2 — Supplementary file2 (TIF 2617 KB) [file 10911_2021_9508_MOESM2_ESM.tif]

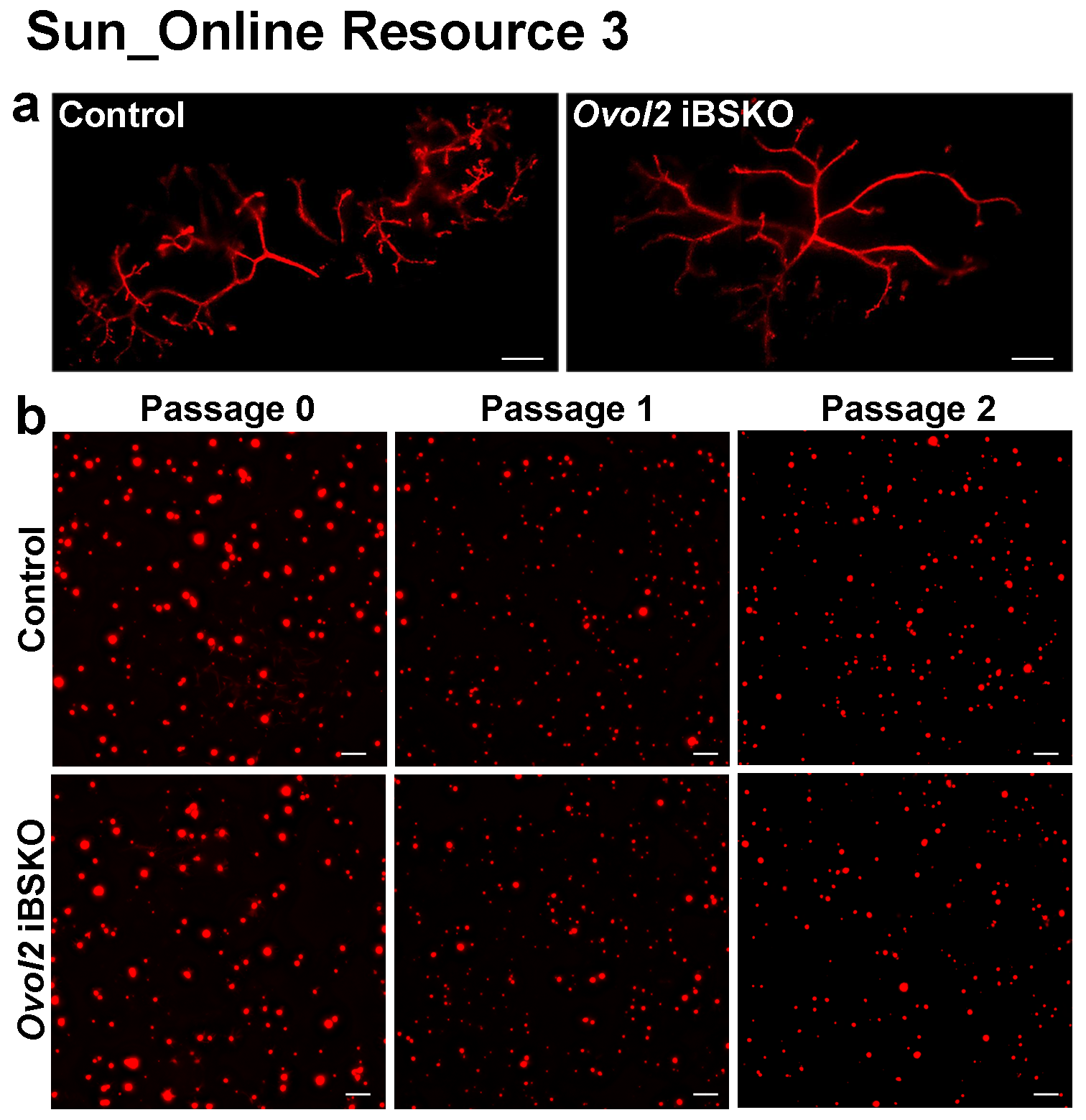

Supplement: Supplementary file 3 — Supplementary file3 (TIF 8468 KB) [file 10911_2021_9508_MOESM3_ESM.tif]
